# Optimizing the predictive validity of NIRS kinetic profiles to predict aerobic capacity from a resting skeletal muscle occlusion test

**DOI:** 10.1113/EP092899

**Published:** 2025-08-22

**Authors:** Heru Syarli Lesmana, Kyohei Marume, Justin S. Lawley

**Affiliations:** ^1^ Department of Sport Science University of Innsbruck Innsbruck Austria; ^2^ Department of Sport Coaching Universitas Negeri Padang Padang Indonesia; ^3^ Department of Cardiovascular National Cerebral and Cardiovascular Center Suita Japan

**Keywords:** muscle oxygenation, peak flow, reactive hyperaemia, ${\dot{V}}_{O_{2}\max}$

## Abstract

Measuring and monitoring individual cardiorespiratory fitness through a valid and accessible non‐exhaustive surrogate is required. Techniques measuring haemodynamics have shown promise, and this study aimed to optimize the predictive validity of these approaches alongside developing predictive equations. In a two‐study design, 8 (pilot study) and 30 (confirmation study) healthy adults completed exercise testing to assess maximal oxygen consumption (${\dot{V}}_{O_{2}\max}$) and an ischaemic occlusion test on the upper thigh to assess superficial femoral blood flow from ultrasonography and skeletal muscle oxygenation (SmO_2_) by near‐infrared spectroscopy (NIRS) before, during and post‐cuff release. In study 1, treadmill running and a 5‐min 220 mmHg ischaemic cuff pressure were performed, whereas in study 2, cycling ergometry and a 3‐min 300 mmHg cuff pressure were applied. In study 1 and study 2, _abs_ ${\dot{V}}_{O_{2}\max}$ and _rel_ ${\dot{V}}_{O_{2}\max}$ were correlated to peak blood flow post‐cuff occlusion (*r* = 0.57–0.84, all *P *< 0.01). In study 2, several NIRS based metrics of post‐occlusive reactive hyperaemia were strongly correlated with _abs_ ${\dot{V}}_{O_{2}\max}$ and _rel_ ${\dot{V}}_{O_{2}\max}$ (all *P *< 0.001). Moreover, the magnitude of oxygen desaturation during the cuff occlusion was highly significantly related to both _abs_ ${\dot{V}}_{O_{2}\max}$ and _rel_ ${\dot{V}}_{O_{2}\max}$ (all *P *< 0.001). As an example, the SmO_2_ desaturation slope was strongly associated with _abs_ ${\dot{V}}_{O_{2}\max}$ (*r* = −0.74, *P *< 0.001). Finally, intercorrelations between the rate of SmO_2_ desaturation during cuff occlusion and the rate of SmO_2_ reoxygenation and peak skeletal muscle blood flow post‐cuff occlusion were observed (*P *< 0.01). An ischaemic‐based test of skeletal muscle haemodynamic profiles could potentially be used to predict ${\dot{V}}_{O_{2}\max}$ and estimate a person's fitness.

## Abstract (highlights)

- **What is the central question of this study?**
  This study aimed to optimize the predictive validity of VO2max through simultaneous ultasonography and NIRS to measure metrics of skeletal muscle hemodynamics during and post a period of limb ischemia alongside developing predictive equations.
- **What is the main finding and its importance?**
  Peak muscle blood flow and NIRS muscle reoxygenation kinetics post a 3‐min period of 300 mmHg limb ischaemia are correlated to oxygen consumption (_rel_ ${\dot{V}}_{O_{2}\max}$ and _abs_ ${\dot{V}}_{O_{2}\max}$) and peak muscle blood flow and the NIRS reoxygenation kinetics are dependent on the degree of muscle ischaemia during cuff occlusion. These data were used to develop a comprehensive set of equations to predict ${\dot{V}}_{O_{2}\max}$ from resting state skeletal muscle blood flow and NIRS kinetic profiles.

## 1 INTRODUCTION

Exercise training induces central adaptations, including increased cardiac output, plasma volume and red cell mass, ultimately enhancing oxygen transport (Astorino et al., 2017; Holloszy & Coyle, 1984; Schmidt & Prommer, 2008). Exercise training also drives peripheral adaptations, such as improved capillary density, vascular function and mitochondrial efficiency (Holloszy & Coyle, 1984; Mølmen et al., 2025; Prior et al., 2003). Improvements in these physiological processes can be collectively estimated by measuring an individual's maximal oxygen consumption (${\dot{V}}_{O_{2}\max}$), which is a critical indicator of aerobic capacity and widely employed to evaluate training efficacy in athletic populations and functional capacity in the elderly. However, the assessment of ${\dot{V}}_{O_{2}\max}$ does not separate central from peripheral adaptations and requires exhaustive exercise testing, which is resource‐intensive and impractical for athletes in remote locations relative to the primary performance centre and patients with cardiorespiratory limitations. Consequently, there is a demand for accessible, non‐exhaustive methods to monitor changes in ${\dot{V}}_{O_{2}\max}$ effectively (Akalan et al., 2004; Lee & Zhang, 2021; Zugck et al., 2000).

Previous research has observed linear relationships between skeletal muscle physiology (i.e., muscle capillary density; Robbins et al., 2011) and/or mitochondrial function (Gifford et al., 2016; Jacobs & Lundby, 2012; Larson‐Meyer et al., 2000; Tonkonologi & Sahlin, 1997; Zoll et al., 2002) and an individual's ${\dot{V}}_{O_{2}\max}$. While these techniques are complicated to perform, costly and invasive, they lay the foundation that the skeletal muscle architecture and function are related to an individual's overall level of fitness (i.e., training status). One simple and non‐invasive solution to estimate skeletal muscle vascular function is the well‐established technique of limb ischaemia and post‐ischaemic reactive hyperaemia. Simply, this technique involves a brief (2–5 min) period of limb occlusion followed by rapid cuff deflation to monitor post‐occlusive reactive hyperaemia. To estimate oxyhaemoglobin and deoxyhaemoglobin in the skeletal muscle microcirculation, duplex ultrasonography has become the research and clinical standard due to its ability to quantify absolute limb blood flow and the magnitude of reactive hyperaemia (Philpott & Anderson, 2007; Rosenberry & Nelson, 2020). Indeed, previous research has shown that peak superficial femoral artery blood flow post‐ischaemia is related (Gifford et al., 2020, *r* = 0.56; Rasica et al., 2022, *r* = 0.65) to an individual's ${\dot{V}}_{O_{2}\max}$. However, this technique is highly user‐dependent, requires an extensive period of training, and is subject to movement artefacts during cuff occlusion and release. An alternative, simplified approach to measure haemodynamic responses to a cuff occlusion test is the use of near‐infrared spectroscopy (NIRS). By measuring the different absorbance wavelengths of near‐infrared light, NIRS technology can indirectly estimate the concentration of both oxygenated and deoxygenated haemoglobin/myoglobin. Thereafter, an integrated metric of muscle oxygen saturation (SmO_2_) is typically calculated as the quotient of oxygenated and deoxygenated haemoglobin/myoglobin. While classically the rate of muscle resaturation is used as an index of muscle oxygen saturation perfusion post‐occlusion, several metrics and normalization procedures can be applied (Beever et al., 2020; Gerovasili et al., 2010; Koutlas et al., 2023; Rasica et al., 2022; Soares et al., 2017).

From a scientific perspective these procedures typically try to account for different baseline values or the depth of desaturation during the occlusion period. However, from an applied perspective, many of these indices show a high level of collinearity (Rasica et al., 2022). As such, it is likely that several of these indices may be used interchangeably to predict a particular outcome variable and some metrics may prove more easily quantifiable and reliable than others. As an example, a previous study identified that the rate of desaturation during cuff occlusion (i.e., the hypoxic stimulus) was related to the rate of reoxygenation post‐cuff release, and when the degree of desaturation is controlled, differences in reoxygenation between young and the elderly become less apparent (Rosenberry et al., 2018, 2019). Applying this same concept, a recent study (Rasica et al., 2022) showed that the desaturation rate during cuff occlusion is correlated to the NIRS reperfusion rate and that the NIRS reperfusion rate is correlated to peak blood flow measured by ultrasonography. Ultimately while not measured previously, it is likely that rate of muscle oxygen desaturation (recently reported for the deoxygenated haemoglobin slope; Koutlas et al., 2023) is also predictive of an individual's ${\dot{V}}_{O_{2}\max}$ and unlike peak blood flow from ultrasound (typically three heart beats) and the reperfusion rate (10 s) is not limited to short time windows with high error probabilities and is less sensitive to the duration of cuff occlusion (Iannetta et al., 2019; Rosenberry et al., 2018).

Therefore, to develop predictive equations for ${\dot{V}}_{O_{2}\max}$, this study utilized simultaneous ultrasonography and NIRS to measure metrics of skeletal muscle haemodynamics during and post a period of limb ischaemia and thereafter correlating these metrics to the measured ${\dot{V}}_{O_{2}\max}$ by cardiopulmonary exercise testing. Importantly, to obtain a realistic appraisal of this approach in an applied setting, classic research procedures such as skin tissue thickness or blood volume correction, and highly standardized NIRS placement were not performed.

## 2 METHODS

The study was performed to the standards set out by the *Declaration of Helsinki*, except for registration in a database, and was approved by the ethics committee of the University of Innsbruck (No. 34/2018). Written informed consent was obtained from all participants following detailed verbal explanations of information regarding all potential risks, the study's requirements and objectives to ensure awareness of the risks of their involvement.

### 2.1 Participants

Study 1 (pilot study) enrolled eight participants, consisting of six males (age, 29.5 ± 3.1 years; height, 1.78 ± 6.3 m; weight, 72.1 ± 6.5 kg) and two females (age, 28.5 ± 4.9 years; height, 1.71 ± 5.9 m; weight, 63.7 ± 4.5 kg) and study 2 enrolled a total of 30 healthy participants, 16 males (age, 26.4 ± 3.6 years; height, 1.82 ± 10.9 m; weight, 76.5 ± 13.2 kg) and 14 females (age, 25.7 ± 4.8 years; height, 1.70 ± 7.8 m; weight, 65.3 ± 12.3 kg). Participants were students from the University of Innsbruck recruited via personal communication (see Table 1 for participant characteristics from both protocols). All participants were healthy and free from known cardiovascular, respiratory, metabolic diseases, and medications that would affect their haemodynamic responses to exercise. The female menstrual cycle phase was not controlled.

**TABLE 1:** Characteristics of participants in study 1 and study 2.

Characteristic	Pilot study 1	Confirmation study 2	
	*n* = 8 (2 female, 6 male)	*n* = 30 (14 female, 15 male)	
Age (years)	29.2 ± 3.2	26.1 ± 4.1	
Height (cm)	176.5 ± 6.64	176.1 ± 11.2	
Weight (kg)	70.1 ± 6.98	70.9 ± 13.8
BMI (kg m^−2^)	22.5 ± 2.1	22.7 ± 2.1	
HR (bpm)	54 ± 10	67 ± 8.7	
Systolic blood pressure (mmHg)	120 ± 12.6	125 ± 9.3	
Diastolic blood pressure (mmHg)	71 ± 9.6	78 ± 10.5	

Data are presented as means ± SD. Abbreviations: BMI, body mass index; HR, heart rate.

### 2.2 Experimental protocol

**FIGURE 1:**
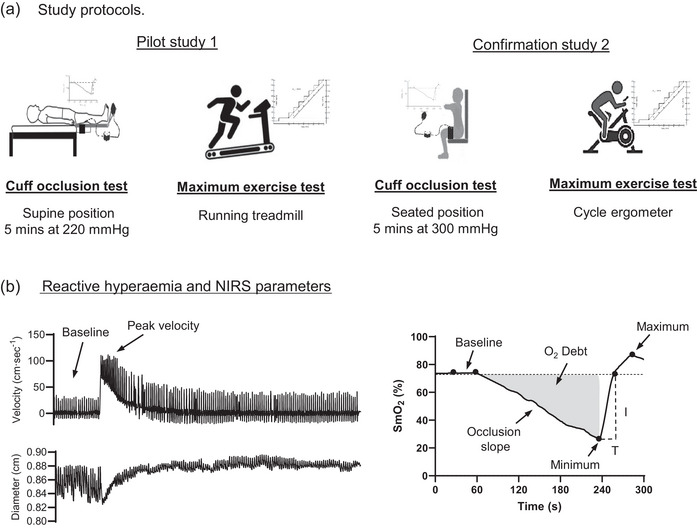
(a) Systematic representation of the study protocols and data processing of ultrasound and NIRS based measures of skeletal muscle. (b) Examples of peak blood flow (i.e., reactive hyperaemia) and NIRS based metrics during and post‐ischaemic cuff test.

In both studies, participants attended the laboratory having abstained from alcohol, caffeine, smoking and exercise for 12 h and having consumed a light meal 4 h prior to testing (Harris et al., 2010). Participants attended the laboratory on two occasions (Figure 1a). On the first visit, after obtaining measurements including height (cm), weight (kg) and blood pressure (mmHg), the cuff occlusion test was performed. On the second visit, participants performed a graded cardio‐pulmonary exercise test to volitional exhaustion.

#### 2.2.1 Cuff occlusion test

A pressure cuff (Hokanson CC17, Bellevue, WA, USA) sized 18 × 108 cm was connected to a blood pressure monitor (Heine Gamma M‐00.09.240, Herrsching, Germany) and placed at the inguinal crease of the right upper thigh regardless of leg dominance.

Study 1: After resting in the supine position for 20 min to achieve haemodynamic stabilization, a 2‐min baseline period was recorded. Thereafter, the thigh cuff was inflated for 5 min at a pressure of 220 mmHg, the participant was instructed to relax the right leg, and the cuff was rapidly deflated. The participant subsequently remained still with the right leg relaxed for 3 min (Bleeker et al., 2005).

Study 2: The procedure was identical except that the participant was seated on a padded chair with their lower leg hanging, and it was decided to maintain a standardized cuff pressure of 300 mmHg for 3 min. All haemodynamic data were recorded continuously during the test except for blood pressure. Only participants from study 2 were used to develop predictive equations.

#### 2.2.2 Maximum exercise test

A quiet room was provided for the test with a temperature between 20°C and –25°C. A spirometric system (Oxycon Pro; CareFusion GmbH, Hoechbach, Germany) was utilized to record breath‐by‐breath gas analysis continuously throughout the exercise tests in studies 1 and 2 and was calibrated prior to each measurement. A Bluetooth chest belt (Wear Link; Polar, Kempele, Finland) was attached to monitor heart rate (HR) and transmitted to the spirometric device.

Study 1: The maximum rate of oxygen consumption during running was measured during a graded running test on a treadmill (HP cosmos Pulsar, Nussdorf, Traunstein, Germany). Participants initially ran at a speed of 8 km h^−1^ with a ∼1% incline and then, each minute, the speed was increased by 1 km h^−1^ until 12 km h^−1^; thereafter, the incline was increased by ∼1% every 30 s until participants reached volitional exhaustion.

Study 2: The maximum rate of oxygen consumption during cycling was measured during an incremental maximal exercise test on a cycling ergometer (Cyclus 2, Avantronic Richter, Leipzig, Germany). Participants started cycling at 100 W with a 5 W increase every 15 s until participants reached volitional exhaustion or the minimum cadence of 85 rpm could no longer be maintained.

### 2.3 Measurements

#### 2.3.1 Haemodynamics

An electrocardiogram was used to monitor HR continuously. An electro‐sphygmomanometer (Tango; SunTechMedical Instruments Inc, NC, USA) measured systolic and diastolic arterial blood pressure from the left arm. Mean arterial pressure was determined by using the formula: [(systolic blood pressure) + 2 × (diastolic blood pressure)]/3.

#### 2.3.2 Superficial femoral blood velocity and artery diameter

Study 1: Superficial femoral artery blood flow (sFBF) velocity and diameter were measured using a 9‐MHz linear‐array transducer continuously via Duplex ultrasonography (iE33; Philips, Best, Netherlands).

Study 2: sFBF velocity and diameter were measured using a 15–4 MHz transducer (15L4 Smart MarK™, Terason, MA, USA) via Duplex ultrasonography (Usmart 3300 NexGen Ultrasound; Teratech Corporation, Burlington, MA, USA).

In both studies, an insonation angle of 60° was used to record the intensity‐weighted time‐averaged mean blood velocity with the sample volume encompassing the entire vessel lumen. The measurement was recorded using the Camtasia Recorder (Camtasia Recorder 8; TechSmith, East Lansing, MI, USA) and analysed offline using custom software. The algorithm used to measure diameter has previously been validated (Coolbaugh et al., 2016) and used by our group to measure the blood flow in different positions of the legs while the upper part of the body is in the supine position (Marume et al., 2023) and changes in femoral diameter associated with exercise (Amin et al., 2021). Experienced sonographers (K.M. and H.S.L.) conducted all ultrasound measurements, and a skin marker was used to maintain probe placement in all measurements.

#### 2.3.3 SmO_2_ by NIRS

An NIRS sensor (NIRO 200, Hamamatsu Photonics, Hamamatsu, Japan) was attached below the cuff on the most prominent part of the vastus lateralis. SmO_2_ (oxy‐haemoglobin/total‐haemoglobin × 100) was recorded continuously at a sampling frequency of 6 Hz, with the method of spatial resolved spectroscopy. The NIRS had three wavelengths (775, 810 and 850 nm) and contained two detectors located at a mean distance of 4 cm from the emitting source. A ‘black‐out’ fabric and bandage were placed over the sensor to omit light artifacts and stabilize the sensor.

### 2.4 Data analysis

#### 2.4.1 Reactive hyperaemia

The baseline artery diameter and blood velocity were measured 1 min before cuff inflation and for 3 min post‐cuff release. Femoral blood flow was determined through previously explained methods (Amin et al., 2021; Hanson et al., 2020) and expressed in absolute terms as mL min^−1^(Figure 1b). Post‐cuff occlusion, several metrics were  obtained to measure reactive hyperaemia and assess microvascular dilator function: (1) peak blood flow is identified as the highest value over three consecutive heart beats; (2) also as the difference in blood flow between baseline and the peak value (Δ sFBF baseline–peak) in an attempt to correct for any baseline differences between individuals or testing sessions; and (3) total blood flow is obtained by measuring the area under the curve over 100 s from when the cuff was released, subtracting baseline blood flow (Iwamoto et al., 2018). On some occasions (*n* = 10), sBFB could not be insonated within a reasonable time frame (typically 1–2 s) post‐cuff release due to the movement artifacts created by cuff deflation. As peak blood flow occurs very rapidly, these individuals were excluded from the analyses.

#### 2.4.2 Reactive hyperaemia SmO_2_

The NIRS device was linked to an analog‐to‐digital converter (Powerlab: ADInstruments, Oxford, UK) with a data sampling rate of 400 Hz. The data were displayed on LabChart (LabChard 8; ADInstruments) and analysed offline (Figure 1b). Several SmO_2_ parameters were calculated in order to identify the best predictor of ${\dot{V}}_{O_{2}\max}$. Relative rate of muscle reoxygenation, that is, the time taken to reach the baseline value (*R*_bl_) and peak reoxygenation (*R*_peak_), was calculated post‐cuff occlusion. Moreover, the reoxygenation rate over 10 s post‐cuff deflation (i.e., reperfusion window; Rep 10 s) was calculated based on previous methods (Soares et al., 2019). The increment or delta oxygenation (*I*) and time constant of muscle reoxygenation (τ) were calculated by multiplying by 0.63. Subsequently, the relative rate of muscle reoxygenation (*R*) could be calculated with the equation: *R* = *I*/τ (Azevedo et al., 2016; Ding et al., 2001; Koutlas et al., 2023). Moreover, *R*_bl_ was calculated as the time taken for SmO_2_ to return to baseline (*I*_bl_/τ_bl_), and *R*_peak_ was calculated as the relative time constant to reach the peak SmO_2_ post‐cuff occlusion (*I*_peak_ /τ_peak_). Slope and integral data were calculated using LabChart software collected from the occlusion period. Finally, the SmO_2_ area under the curve (AUC_2min_) was calculated from the time of cuff release to 2 min post‐cuff occlusion and also for the occlusion period (AUC occlusion) using the trapezoidal rule (Rosenberry et al., 2018). Data were excluded (study 1: *n* = 1 and study 2: *n* = 5) if the NIRS signal was lost in the occlusion period or post‐cuff release.

### 2.5 Power calculation

A power calculation was performed based on pilot data from study 1. From these data, we assumed a minimum correlation between ${\dot{V}}_{O_{2}\max}$ and NIRS indices of *r* = 0.68, with an α level of 0.05 and 95% statistical power, a minimum sample size was estimated at 18 participants (G*power v3.1.9.4).

### 2.6 Statistical analyses

Pearson's linear correlation was applied to examine the relationships between ${\dot{V}}_{O_{2}\max}$ and indices of RH from both femoral blood flow and SmO_2._ Moreover, Pearson's correlations were performed between the peak blood flow obtained from ultrasonography and two of the primary NIRS based indices of muscle oxygenation. Correlation coefficients were classified following the recommendations as follows: very weak <0.2, weak <0.40, moderate <0.60, strong <0.80, very strong >0.80 (Evans, 1996). All statistical analyses were performed using SPSS Statistics (Version 25, IBM Corp., Armonk, NY, USA) and the graphs created on Prism (Version 8.4.2, GraphPad Software Inc., San Diego, CA, USA). Linear regression was performed to provide the predictive equations. Data are presented as means ± standard deviation (SD), and α was set at *P *≤ 0.05.

## 3 RESULTS

### 3.1 Maximal exercise test and post‐occlusion tests

Mean data from the maximal exercise test and post‐occlusion tests (i.e., blood flow and NIRS indices of muscle reoxygenation) from study 1 and study 2 are presented in Table 2.

**TABLE 2:** Exercise parameters, post‐occlusion blood flow and NIRS parameters in study 1 and study 2.

	Pilot study 1	Confirmation study 2
_abs_ ${\dot{V}}_{O_{2}\max}$ (mL min^−1^)	3939 ± 920	3989 ± 902
_rel_ ${\dot{V}}_{O_{2}\max}$ (mL kg min^−1^)	55.6 ± 9.9	56.1 ± 7.1
HR_max_ (bpm)	189 ± 6.5	187 ± 8.7
PPO (w)	—	315 ± 67.5
sFBF baseline (mL min^−1^)	89.71 ± 29.8	94.83 ± 38.8
RH peak flow (mL min^−1^)	1203 ± 196.7	1224 ± 400.3
Rep 10 s (% s^−1^)	2.77 ± 1.08	1.68 ± 0.81
*R*_bl_ (% s^−1^)	3.42 ± 1.46	2.55 ± 1.17
*R*_peak_ (% s^−1^)	2.77 ± 1.08	2.05 ± 0.85
AUC_2min_ (%)	3909 ± 1072	2347 ± 778.5

Data are presented as means ± SD. Abbreviations: _abs_ ${\dot{V}}_{O_{2}\max}$ , absolute maximum oxygen uptake; AUC_2min_, area under the curve over 2 min post‐cuff release; HR_max_, maximum heart rate; PPO, peak power output; *R*_bl_, relative rate of muscle oxygenation back to baseline values; _rel_ ${\dot{V}}_{O_{2}\max}$, relative maximum oxygen uptake; Rep 10 s, reperfusion rate over 10 s; RH, reactive hyperaemia; *R*_peak_, relative rate of muscle oxygenation to reach peak values; sFBF, superficial femoral blood flow; SmO_2_, muscle oxygen saturation.

#### 3.1.1 Pilot Study 1

**FIGURE 2:**
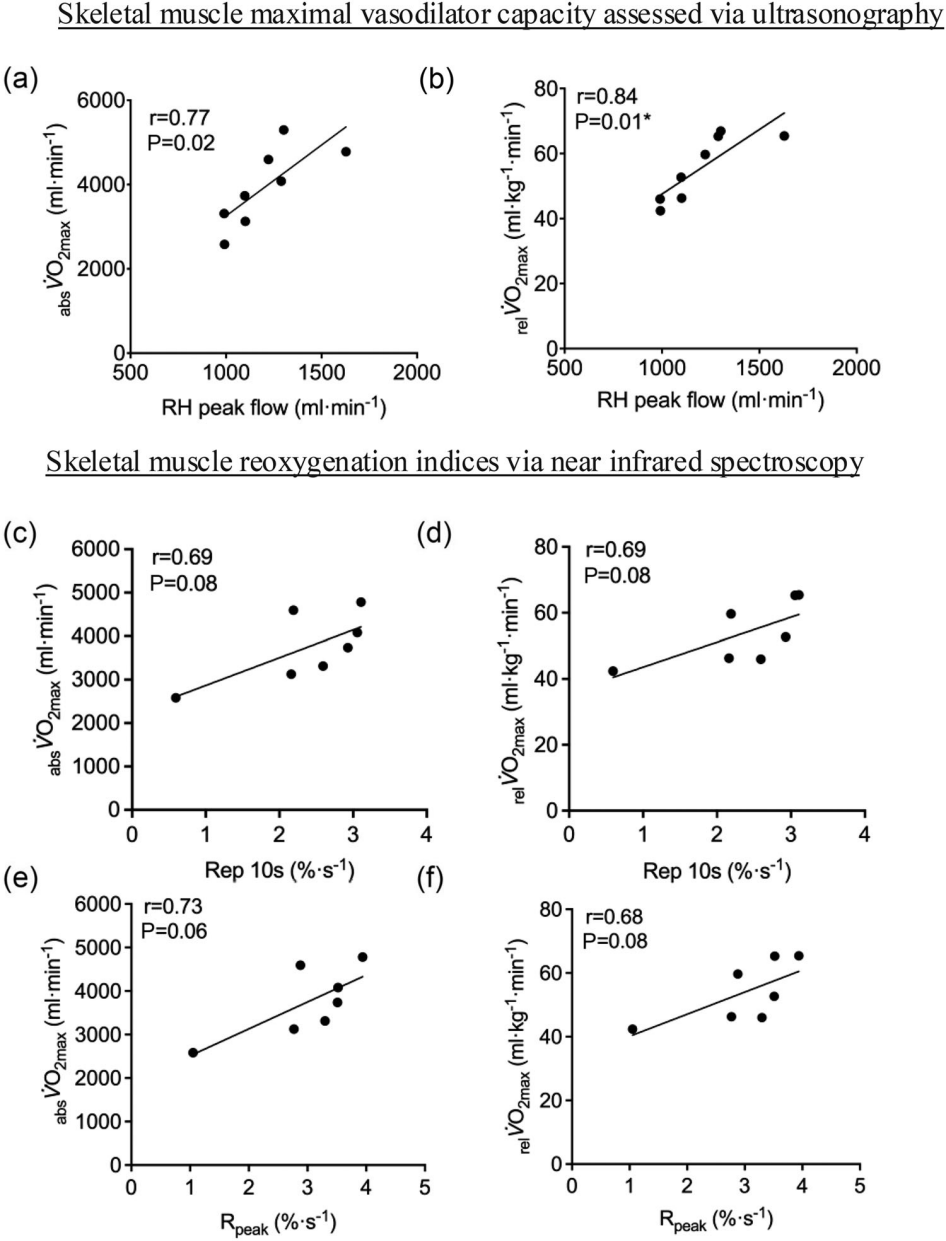
Pilot data showing potential linear correlations between ${\dot{V}}_{O_{2}\max}$ and ultrasound and near‐infrared spectroscopy‐based metrics of skeletal muscle relative hyperaemia. (a, b) Reactive hyperaemia (RH) peak superficial femoral artery blood flow (*n* = 8) after 3 min of occlusion (250 mmHg) correlated to absolute (_abs_ ${\dot{V}}_{O_{2}\max}$, a) and relative (_rel_ ${\dot{V}}_{O_{2}\max}$, b) maximal oxygen uptake. (c–f) Two indices of muscle reoxygenation (*n* = 7), that is, the muscle reoxygenation rate over 10 s (Rep 10 s) and relative muscle oxygenation to peak (*R*_Peak_), correlated to absolute (_abs_ ${\dot{V}}_{O_{2}\max}$, c, d) and relative (_rel_ ${\dot{V}}_{O_{2}\max}$, e, f). See Section 3 for other indices.

In this pilot sample (*n* = 8), a strong statistical correlation was observed between the RH peak flow and the _rel_ ${\dot{V}}_{O_{2}\max}$ (*r* = 0.84, *P* = 0.01) and _abs_ ${\dot{V}}_{O_{2}\max}$ (*r* = 0.77, *P* = 0.02) (see Figure 2). Moreover, the ΔsFBF baseline–peak (_rel_ ${\dot{V}}_{O_{2}\max}$, *r* = 0.80, *P* = 0.02; _abs_ ${\dot{V}}_{O_{2}\max}$, *r* = 0.72, *P* = 0.04) and the RH AUC (_rel_ ${\dot{V}}_{O_{2}\max}$, *r* = 0.68 *P* = 0.06; _abs_ ${\dot{V}}_{O_{2}\max}$, *r* = 0.73, *P* = 0.04) were correlated to maximal oxygen uptake (see Table 3). NIRS based indices of muscle reoxygenation showed promise with strong correlation coefficients (i.e., *r* = 0.68–0.75), but statistical significance varied due to either skewed data, misleading statistical significance, or a limited distribution in NIRS data (see Figure 2 and Table 3).

**TABLE 3:** Linear regression between oxygen uptake and post‐occlusion blood flow and NIRS parameters in study 2.

		*R^2^*	SEE	95% CI		
				Slope	*Y*‐intercept	*X*‐intercept
RH peak flow (mL min^−1^)	_abs_ ${\dot{V}}_{O_{2}\max}$ (mL min^−1^)	0.64	551	1.59, 3.61	−372.1, 2017	−1257, 103.8
	_rel_ ${\dot{V}}_{O_{2}\max}$ (mL kg min^−1^)	0.35	5.99	0.0047, 0.0267	23.23, 49.25	−10,292, −874.1
*R*_peak_ (% s^−1^)	_abs_ ${\dot{V}}_{O_{2}\max}$ (mL min^−1^)	0.56	606	495, 1096	1708, 3029	−6.04, −1.57
	_rel_ ${\dot{V}}_{O_{2}\max}$ (mL kg min^−1^)	0.37	5.75	2.16, 7.86	39.58, 52.13	−23.8, −5.072
Rep 10 s (% s^−1^)	_abs_ ${\dot{V}}_{O_{2}\max}$ (mL min^−1^)	0.56	608	526, 1168	2072, 3210	−6.01, −1.8
	_rel_ ${\dot{V}}_{O_{2}\max}$ (mL kg min^−1^)	0.38	5.70	2.44, 8.46	42.06, 52.73	−21.33, −5.02
Slope occlusion (% s^−1^)	_abs_ ${\dot{V}}_{O_{2}\max}$ (mL min^−1^)	0.54	623	−26,500, −11,469	1544, 2999	0.05896, 0.2584
	_rel_ ${\dot{V}}_{O_{2}\max}$ (mL kg min^−1^)	0.41	5.57	−196.3, −61.80	37.9, 50.91	0.1945, 0.8177

Abbreviations: _abs_ ${\dot{V}}_{O_{2}\max}$, absolute maximum oxygen uptake; Cl, confidence interval; *R^2^*, coefficient of determination; _rel_ ${\dot{V}}_{O_{2}\max}$, relative maximum oxygen uptake; Rep 10 s, reperfusion rate over 10 s; RH, reactive hyperaemia; *R*_peak_, relative rate of muscle oxygenation to reach peak values; SEE, standard error of the estimate.

#### 3.1.2 Study 2

**FIGURE 3:**
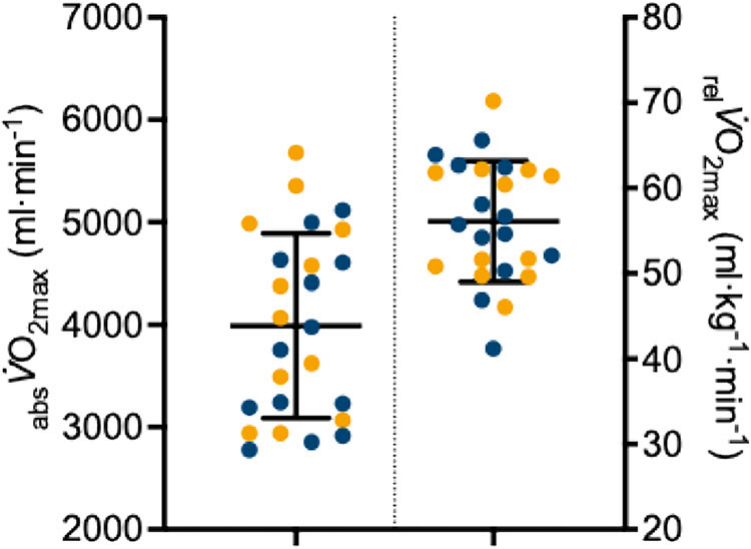
Distribution of absolute (_abs_ ${\dot{V}}_{O_{2}\max}$) and relative (_rel_ ${\dot{V}}_{O_{2}\max}$) cardiorespiratory fitness in the confirmation sample (*n* = 30). Orange symbols, male participants; blue symbols, female participants.

**FIGURE 4:**
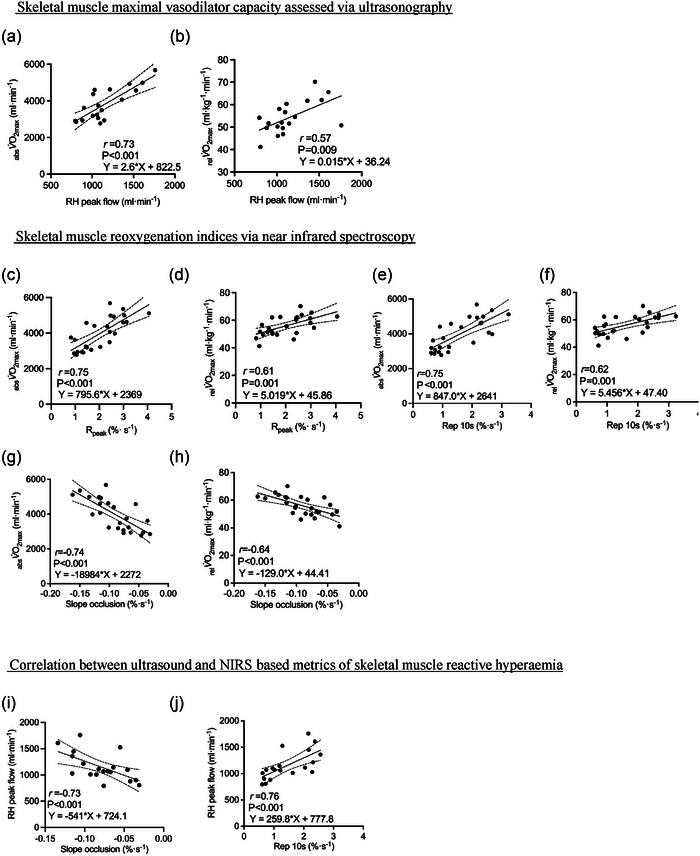
Confirmation study confirming linear correlations between ${\dot{V}}_{O_{2}\max}$ and ultrasound and near‐infrared spectroscopy‐based metrics of skeletal muscle relative hyperaemia. (a, b) Reactive hyperaemia (RH) peak superficial femoral artery blood flow (*n* = 20) after 3 min of occlusion (300 mmHg) correlated to absolute (_abs_ ${\dot{V}}_{O_{2}\max}$, a) and relative (_rel_ ${\dot{V}}_{O_{2}\max}$, b) maximal oxygen uptake. (c–h) Two indices of muscle reoxygenation (*n* = 25), that is, relative muscle oxygenation to peak (*R*_Peak_) and the muscle reoxygenation rate over 10 s (Rep 10 s), correlated to absolute (_abs_ ${\dot{V}}_{O_{2}\max}$, c, d) and relative (_rel_ ${\dot{V}}_{O_{2}\max}$, e, f). Two metrics estimating the degree of hypoxic stress imposed by the occlusion, that is, Slope occlusion rate and the slope integral correlated to absolute (_abs_ ${\dot{V}}_{O_{2}\max}$, g) and relative (_rel_ ${\dot{V}}_{O_{2}\max}$, h). (i, j) Correlations between one metric estimating the hypoxic stress caused by the occlusion and the degree of post‐occlusion reactive hyperaemia (*n* = 20) (i) and the magnitude of post‐occlusive hyperaemia from ultrasound and muscle reoxygenation rate via NIRS (j). See the tables and Section 3 for other indices. Only participants from study 2 were used to develop predictive equations. The dashed lines represent the 95% confidence interval of the model.

In the follow‐up confirmation study, there was a wide range in cardiorespiratory fitness in both men and women (see Figure 3) and we again observed strong statistical correlations between the RH peak flow and _rel_ ${\dot{V}}_{O_{2}\max}$ (*r* = 0.57, *P* = 0.009) and _abs_ ${\dot{V}}_{O_{2}\max}$ (*r* = 0.73, *P *< 0.001) (see Figure 4, *n* = 20). Moreover, the ΔsFBF baseline–peak was correlated to _rel_ ${\dot{V}}_{O_{2}\max}$ (*r* = 0.58, *P* = 0.07) and _abs_ ${\dot{V}}_{O_{2}\max}$ (*r* = 0.72, *P *< 0.01) (see Table 3). For NIRS based metrics of muscle reoxygenation, we obtained a greater distribution of scores in the follow‐up study with an increase in sample size (*n* = 25). As such, positive statistical correlations were observed between _rel_ ${\dot{V}}_{O_{2}\max}$ (Rep 10 s, *r* = 0.61, *P* = 0.001; *R*_peak_, *r* = 0.61, *P* = 0.001; AUC_2min_, *r* = 0.60, *P* = 0.001; *R*_bl_, *r* = 0.59, *P* = 0.002) and _abs_ ${\dot{V}}_{O_{2}\max}$ (Rep 10 s, *r* = 0.75, *P *< 0.001; *R*_peak_, *r* = 0.75, *P *< 0.001; AUC_2min_, *r* = 0.73, *P *< 0.001; *R*_bl_, *r* = 0.77, *P *< 0.001) and all parameters of SmO_2_ post‐cuff release. Moreover, three metrics estimating the hypoxaemic stress incurred during the cuff occlusion (slope, AUC and integral) were similarly correlated with _rel_ ${\dot{V}}_{O_{2}\max}$ (slope, *r* = −0.64, *P *< 0.001; AUC, *r* = −0.68, *P* = 0.004; Integral, *r* = −0.56, *P* = 0.004; and _abs_ ${\dot{V}}_{O_{2}\max}$ (slope, *r* = −0.74, *P *< 0.001; AUC, *r* = −0.74, *P *< 0.001; Integral, *r* = −0.67, *P *< 0.001) (see Table 4). The predictive equations for the _abs_ ${\dot{V}}_{O_{2}\max}$ showed RH peak flow had *R* ^2^ = 0.64 the standard error of the estimate (SEE) = 551 (1.59, 3.61), *R*_peak_ had *R* ^2^ = 0.56 SEE = 606 (495, 1096), Rep 10 had *R* ^2^ = 0.56 SEE = 608 (526, 1168) and slope occlusion had *R* ^2^ = 0.54 SEE = 6234 (−26,500, −11,469). For the predictive _rel_ ${\dot{V}}_{O_{2}\max}$ equations RH peak flow had *R* ^2^ = 0.35 SEE = 5.99 (0.0047, 0.027), *R*_peak_ had *R* ^2^ = 0.37 SSE = 5.75 (2.16, 7.86), Rep 10 had *R* ^2^ = 0.38 SEE = 5.7 (2.44, 8.46) and slope occlusion had *R* ^2^ = 0.41 SSE = 5.57 (−196.3, −61.80) (see Figure 4 for all predictive equations and Table 3).

**TABLE 4:** Linear correlation between maximal oxygen uptake and post‐occlusion blood flow and NIRS parameters in study 1 and study 2.

	Pilot study 1		Confirmation study 2	
	_abs_ ${\dot{V}}_{O_{2}\max}$ (mL min^−1^)	_rel_ ${\dot{V}}_{O_{2}\max}$ (mL kg min^−1^)	_abs_ ${\dot{V}}_{O_{2}\max}$ (mL min^−1^)	_rel_ ${\dot{V}}_{O_{2}\max}$ (mL kg min^−1^)
BF RH				
RH peak flow (mL min^−1^)	*r* = 0.77	*r* = 0.84	*r* = 0.73	*r* = 0.57
	*P* = 0.02	*P* = 0.01	*P *< 0.001	*P* = 0.009
Δ sFBF baseline‐peak (mL min^−1^)	*r* = 0.72	*r* = 0.80	*r* = 0.72	*r* = 0.58
	*P* = 0.04	*P* = 0.02	*P *< 0.001	*P* = 0.007
RH AUC (mL min^−1^)	*r* = 0.73	*r* = 0.68	—	—
	*P* = 0.04	*P* = 0.06	—	—
SmO_2_ RH				
Rep 10 s (% s^−1^)	*r* = 0.69	*r* = 0.69	*r* = 0.75	*r* = 0.61
	*P* = 0.08	*P* = 0.08	*P *< 0.001	*P* = 0.001
*R*_peak_, (% s^−1^)	*r* = 0.73	*r* = 0.68	*r* = 0.75	*r* = 0.61
	*P* = 0.06	*P* = 0.08	*P *< 0.001	*P* = 0.001
AUC_2min_ (%)	*r* = 0.74	*r* = 0.68	*r* = 0.73	*r* = 0.60
	*P* = 0.05	*P* = 0.08	*P *< 0.001	*P* = 0.001
*R*_bl_ (%/s)	*r* = 0.71	*r* = 0.75	*r* = 0.77	*r* = 0.59
	*P* = 0.07	*P* = 0.05	*P *< 0.001	*P* = 0.002
Hypoxaemic stress				
Slope occlusion (% s^−1^)	—	—	*r* = −0.74	*r* = −0.64
	—	—	*P *< 0.001	*P *< 0.001
Integral occlusion (% s^−1^)	—	—	*r* = −0.67	*r* = −0.56
	—	—	*P *< 0.001	*P* = 0.004
AUC occlusion (%)	—	—	*r* = −0.74	*r* = −0.68
	—	—	*P *< 0.001	*P* = 0.004

Abbreviations: _abs_ ${\dot{V}}_{O_{2}\max}$, absolute maximum oxygen uptake; AUC occlusion, area under the curve 3 min occlusion period; AUC_2min_, area under the curve over 2 min post‐cuff release, *R*_b_, relative rate of muscle oxygenation back to baseline values; _rel_ ${\dot{V}}_{O_{2}\max}$, relative maximum oxygen uptake; Rep 10 s, reperfusion rate over 10 s; RH, reactive hyperaemia; *R*_peak_, relative rate of muscle oxygenation to reach peak values; sFBF, superficial femoral blood flow; SmO_2_, muscle oxygen saturation.

### 3.2 Relationship between peak blood flow post‐cuff occlusion and NIRS measurements

Peak blood flow post‐occlusion was statistically correlated with both the Rep 10 s (*r* = 0.76, *P *< 0.001), *R*_peak_, *r *= 0.71, *P *< 0.001 and *R*_bl_ (*r* = 0.75, *P *< 0.001). Moreover, peak blood flow post‐occlusion was also statistically correlated with all metrics of hypoxic stress during the cuff occlusion period (slope, *r* = −0.73, *P *< 0.001; AUC, *r* = −0.73, *P *< 0.001; Integral, *r* = −0.76, *P *< 0.001; see Figure 4 and Table 5).

**TABLE 5:** Linear correlation between post‐occlusion blood flow and NIRS parameters in study 2.

	RH peak flow (mL min^−1^)
SmO_2_ RH	
Rep 10 s (% s^−1^)	*r* = 0.76
	*P *< 0.001
*R*_peak_, (% s^−1^)	*r* = 0.71
	*P *< 0.001
*R*_bl_ (% s^−1^)	*r* = 0.75
	*P *< 0.001
Hypoxaemic stress	
Slope occlusion (% s^−1^)	*r* = −0.73
	*P *< 0.001
Integral occlusion (% s^−1^)	*r* = −0.76
	*P *< 0.001
AUC occlusion (%)	*r* = −0.73
	*P *< 0.001

Abbreviations: AUC occlusion, area under the curve 3 min occlusion period; *R*_bl_, relative rate of muscle oxygenation back to baseline values; Rep 10 s, reperfusion rate over 10 s; RH, reactive hyperaemia; *R*_peak_, relative rate of muscle oxygenation to reach peak values; SmO_2_, muscle oxygen saturation.

## 4 DISCUSSION

The goal of the current study was to develop prediction equations for ${\dot{V}}_{O_{2}\max}$ from non‐invasive and non‐exhaustive skeletal muscle haemodynamic profiles. The main findings were that, at rest, a 3‐min period of skeletal muscle ischaemia (300 mmHg) causes a rapid rise in muscle blood flow (assessed via ultrasonography) that is statistically correlated to the measured maximal oxygen consumption (_rel_ ${\dot{V}}_{O_{2}\max}$ and _abs_ ${\dot{V}}_{O_{2}\max}$) during cardiopulmonary exercise testing. Moreover, using the less technically demanding NIRS technique, reperfusion indices post‐cuff release are also correlated to both _rel_ ${\dot{V}}_{O_{2}\max}$ and _abs_ ${\dot{V}}_{O_{2}\max}$. Finally, intra‐correlations were observed between the degree of hypoxic stress during the cuff occlusion period, the measured skeletal muscle hyperaemia, and NIRS based reperfusion rate post‐cuff release. Physiologically these data imply that the degree of muscle ischaemia during cuff occlusion is dependent on the individual's training status (and likely resting skeletal muscle oxidative capacity), whereby a greater degree of skeletal muscle desaturation during cuff occlusion (ischaemic stimulus) causes a greater downstream vasodilatation of the skeletal muscle microvasculature and thus a greater absolute rise in muscle blood flow (hyperaemia) and a faster re‐oxygenation kinetic. Practically, these data imply that while ultrasound provides the best absolute quantification of muscle blood flow during the ischaemic test, the simpler measurement of the occlusion slope and/or re‐oxygenation period by NIRS can be used to predict an individual's ${\dot{V}}_{O_{2}\max}$.

### 4.1 Peak blood flow via ultrasonography

Exercise training causes central adaptations including greater cardiac volumes, left and right ventricular mass (Astorino et al., 2017; Hellsten & Nyberg, 2015), and an elevated plasma volume and red cell mass (Prior et al., 2003; Schmidt & Prommer, 2008). Moreover, improvements in peripheral factors, including skeletal muscle vascular function and skeletal muscle oxidative capacity, are prevalent to maintain high levels of oxygen extraction (Jacobs & Lundby, 2012; Larson‐Meyer et al., 2000; Tonkonologi & Sahlin, 1997; Zoll et al., 2002). For example, previous invasive approaches have clearly identified a linear increase in muscle blood flow and absolute A‐vO_2_diff with increasing exercise intensity, and the greatest ability to increase muscle blood flow is observed in individuals with the highest ${\dot{V}}_{O_{2}\max}$ (Mortensen et al., 2005). A brief period of arterial occlusion induces muscle hypoxia, which triggers profound vasodilatation in the downstream microvasculature. This response is mediated by the interplay of nitric oxide, prostaglandins, potassium and adenosine production (Rosenberry & Nelson, 2020). Following cuff release, blood flow increases rapidly, likely in proportion to the downstream rise in vascular conductance. Consequently, individuals who experience the most substantial hypoxic stimulus during arterial occlusion (i.e., the stimulus for vasodilatation) and posess a more extensive vascular network are expected to exhibit the greatest increase in blood muscle flow upon cuff release. Since skeletal muscle vascular conductance (i.e., vasodilatation) is linearly related to limb ${\dot{V}}_{O_{2}}$ during exercise (Calbet et al., 2004), it is not surprising that blood flow after a period of muscle ischaemia, which likely causes near‐maximal vasodilatation, is related to ${\dot{V}}_{O_{2}\max}$ (Gifford et al., 2020; Pyke et al., 2007; Rasica et al., 2022).

### 4.2 NIRS based metrics of skeletal muscle haemodynamics

In the current study, all NIRS derived metrics were statistically related to ${\dot{V}}_{O_{2}\max}$, and, in general, stronger relationships were observed for _abs_ ${\dot{V}}_{O_{2}\max}$ compared to _rel_ ${\dot{V}}_{O_{2}\max}$. Consistent with previous reports, the NIRS reperfusion rate was strongly correlated with both _abs_ ${\dot{V}}_{O_{2}\max}$ and _rel_ ${\dot{V}}_{O_{2}\max}$ (Rasica et al., 2022). Moreover, three metrics quantifying the rate of oxygen desaturation during the cuff occlusion were strongly correlated with both _abs_ ${\dot{V}}_{O_{2}\max}$ and _rel_ ${\dot{V}}_{O_{2}\max}$. Recently, a similarly strong correlation (*r* = 0.763) was observed between the rate of deoxyhaemoglobin increase during cuff occlusion and _rel_ ${\dot{V}}_{O_{2}\max}$ (Koutlas et al., 2023). To the best of our knowledge, none of the aforementioned studies have reported the regression equations linking NIRS based metrics with ${\dot{V}}_{O_{2}\max}$. Yet, from the current dataset, one interesting observation is that, in general, the regression line crosses the *Y*‐axis (intercept) around an absolute oxygen uptake of ∼2300 mL min^−1^. This means that, in young healthy individuals, it is not possible to predict a ${\dot{V}}_{O_{2}\max}$ less than these calculated values. The underlying cause for this baseline effect is not clear, but it is likely due to the NIRS technique's focus on peripheral vascular function, whereas other central factors, such as cardiac output and red cell mass that can limit ${\dot{V}}_{O_{2}\max}$, are not considered.

### 4.3 Methodological considerations

The current NIRS occlusion technique is simple to apply and requires limited technical skill and post‐processing procedures, as opposed to ultrasonography. The technique consists of a 3–5‐min cuff inflation period at a fixed cuff pressure, which can be applied in an automated fashion or with a simple hand‐held sphygmomanometer. Interestingly, other more complicated techniques that require exercise alongside multiple varying length cuff‐occlusion events and complicated post‐processing procedures that propose to measure post‐exercise skeletal muscle oxidative capacity have been shown to be equally or less predictive of ${\dot{V}}_{O_{2}\max}$ (Beever et al., 2020; Lagerwaard et al., 2021). It is also worth mentioning that classic research procedures such as measuring adipose tissue thickness, normalization factors for total haemoglobin, and precise sensor location were not adhered to in the current study, suggesting this technique may be applied to the more general population.

### 4.4 NIRS device specificity and transferability

Measurements were all performed with a single NIRS device (Hamamatsu NIRO 200). Thus, a major methodological consideration is the transferability of these equations to other devices, especially commercially available devices that are optimized for portability with varying penetration depth (∼15– 20 mm). Future research providing a cross‐platform validation would be warranted.

### 4.5 Limitations

One of the major limitations of the current study is the inclusion of a healthy, well‐trained population. While this population allowed us to develop predictive equations up to a _rel_ ${\dot{V}}_{O_{2}\max}$ of ∼60 mL kg min^−1^ and _abs_ ${\dot{V}}_{O_{2}\max}$ of ∼5.0 L min^−1^, the results cannot be easily extended to a less fit population and/or those with cardiovascular disease. One interesting observation is that a previous study (Rasica et al., 2022) measured an average oxygen desaturation rate of ∼0.05% s^−1^ in young untrained females with a _rel_ ${\dot{V}}_{O_{2}\max}$ of 40.9 mL kg min^−1^ and another study measured an oxygen desaturation rate of ∼0.05% s^−1^ in elderly (74 years) individuals (Rosenberry & Nelson, 2020; Rosenberry et al., 2019). The lowest desaturation rates were also close to ∼0.05% s^−1^ in the current study. While future work is required, it initially seems that the current technique may have a basement effect in young and old individuals. While the current study was not designed to statistically assess potential sex differences, Figure 3 clearly shows an overlap in _rel_ ${\dot{V}}_{O_{2}\max}$ and _abs_ ${\dot{V}}_{O_{2}\max}$ between males and female participants in our study population. Thus, it is highly likely that a previous study suggesting a difference in NIRS kinetics between males and females (Rasica et al., 2022) is due to study‐specific differences in fitness between males and females and not an inherent sex‐based difference. Other technical limitations of this study are that (1) measurements were all performed with a single NIRS device, and it is unknown if the results and predictive equations can be translated to other devices; (2) we only performed a single occlusion test, whereas repeated measurements may improve predictive validity; and (3) the current data suggest the occlusion technique can be used to provide a general guideline to an individual's overall fitness, yet future research is needed to determine if this approach has the sensitivity to detect changes due to structured training.

### 4.6 Conclusion

The main findings of this study were that NIRS kinetic profiles during a cuff occlusion are correlated with an individual's maximal aerobic fitness without the need for exhaustive exercise testing.

## AUTHOR CONTRIBUTIONS

All authors took part in the study's design and concept. Heru Syarli Lesmana and Kyohei Marume performed the measurements and then provided analysed and interpreted data. Justin S. Lawley supervised the study. Heru Syarli Lesmana drafted the manuscript, and all authors reviewed and contributed to the final manuscript. All authors have read and approved the final version of this manuscript and agree to be accountable for all aspects of the work in ensuring that questions related to the accuracy or integrity of any part of the work are appropriately investigated and resolved. All persons designated as authors qualify for authorship, and all those who qualify for authorship are listed.

## CONFLICT OF INTEREST

The authors have no conflict of interest to declare.

## FUNDING INFORMATION

None.

## Data Availability

The data that support the findings of this study are available from the corresponding author upon reasonable request.
